# Comparative Analyses of the Subgingival Microbiome in Chronic Periodontitis Patients with and without Gingival Erosive Oral Lichen Planus Based on 16S rRNA Gene Sequencing

**DOI:** 10.1155/2021/9995225

**Published:** 2021-06-27

**Authors:** Haohao Liu, Huiwen Chen, Yue Liao, Huxiao Li, Linjun Shi, Yiwen Deng, Xuemin Shen, Zhongchen Song

**Affiliations:** ^1^Department of Periodontology, Shanghai Ninth People's Hospital, College of Stomatology, Shanghai Jiao Tong University School of Medicine, Shanghai, China; ^2^Shanghai Key Laboratory of Stomatology & Shanghai Research Institute of Stomatology, National Clinical Research Center for Oral Diseases, Shanghai, China; ^3^Department of Oral Mucosal Diseases, Shanghai Ninth People's Hospital, College of Stomatology, Shanghai Jiao Tong University School of Medicine, Shanghai, China

## Abstract

The aim of the study was to compare the microbiota composition and bacterial diversity of subgingival plaque in chronic periodontitis patients with and without gingival erosive oral lichen planus. The subgingival plaque samples of 20 chronic periodontitis patients with gingival erosive oral lichen planus (CP-OLP group) and 19 chronic periodontitis patients without gingival erosive oral lichen planus (CP group) were analyzed by 16S rRNA gene high-throughput sequencing. Compared with the CP group, the richness and diversity of subgingival plaque microflora in the CP-OLP group decreased significantly. There were some differences between the two groups in the composition of microflora on the levels of phylum and genus. Distributions of *Prevotella* and *Leptotrichia* in the CP-OLP group were significantly lower than those in the CP group. The dominant genera in CP-OLP group were *Pseudomonas* and *Granulicatella*. These results indicated that gingival erosive oral lichen planus may influence the structure and proportion of subgingival plaque microflora.

## 1. Introduction

Periodontitis is characterized by microbially associated, host-mediated inflammation, which leads to damage of periodontal supporting tissues. It is considered one of the primary causes of tooth loss in adults. Recently, many studies have shown that periodontitis is related to the development of oral lichen planus [1–3]. Oral lichen planus (OLP) is an immune-mediated oral and cutaneous inflammatory disease, including a predilection for female gender and middle age [4]. The primary lesions are located in the buccal mucosa, tongue, or gingiva. For some patients, an oral lichen planus lesion is only located in the gingiva [5]. Involvement of gingiva is characterized by intense erythema of the gingiva with desquamation. It may subsequently develop into more serious desquamation, blistering, erosion, or ulceration. Oral lesions in OLP may bleed and be painful. Thus, patients cannot adequately perform oral hygiene habits [6]. Therefore, the severity of OLP would worsen periodontitis symptoms. Long-term bacterial plaque accumulation induces sustained inflammation and affects OLP lesion healing.

Plaque biofilm is the initiating factor of periodontitis. Subgingival plaque biofilm mainly contains G^−^ anaerobic bacteria, which are closely related to the progress of periodontitis [7]. Periodontitis is due to the complex interaction between oral symbiotic microbiota, host susceptibility, and environmental factors such as diet and smoking, which disrupts the balance between bacteria and host and damages the health of periodontal supporting tissues [8]. The etiopathogenesis of OLP remains unclear so far. However, it has been verified to involve microbial infections in the oral cavity [9]. A recent study found a decrease in *Streptococcus* and increases in gingivitis/periodontitis-associated bacteria in OLP lesions; meanwhile, selected oral bacteria damage the physical epithelial barrier and are internalized into epithelial cells or T cells [10]. Ertugrul et al. [11] found that OLP patients have higher levels of infection with periodontal pathogens than non-OLP patients and identified the importance of periodontal pathogenic microorganisms in the progress of periodontal diseases of OLP.

Oral microbiome is extremely complex, and the oral microbial profile may have potential applications for assessing disease risk. However, the profile of subgingival plaque microbiome of erosive gingival oral lichen planus has not been clearly identified. This cross-sectional study is aimed at investigating the periodontal status of gingival erosive oral lichen planus patients and its relationship with subgingival microbiome.

## 2. Methods

### 2.1. Ethics Statement

The study design, protocol, and informed consent were approved by the Institutional Ethics Committee of the Shanghai Ninth People's Hospital Affiliated to Shanghai Jiao Tong University School of Medicine prior to the implementation of the study (No. 2017-434-T330). Written informed consent was obtained from all participants at the first visit.

### 2.2. Study Participant Recruitment

A total of 39 participants were consecutively recruited in the Department of Periodontology, Ninth People's Hospital Affiliated to Shanghai Jiao Tong University School of Medicine, from April 2018 to December 2019 and divided into two groups: gingival erosive oral lichen planus patients with chronic periodontitis (CP-OLP group, 6 males, 13 females, average age 49.5 ± 8.7 years) and patients only with chronic periodontitis (CP group, 8 males, 12 females, average age 45.8 ± 8.0 years).

### 2.3. Inclusion and Exclusion Criteria

Inclusion criteria of the CP group were as follows: (a) aged 18-70 years, (b) a diagnosis of chronic periodontitis (≥30% sites with pocket depth (PD) > 4 mm and clinical attachment loss (CAL)≥3 mm) in accordance with the 1999 International Workshop for a Classification of Periodontal Disease and Conditions [12], and (c) at least 12 natural teeth (except for the third molars).

Inclusion criteria of the CP-OLP group are the following: except for the inclusion criteria of the CP group that are needed to be followed, patients should present (a) oral lesions occurring in the gingiva with white or gray papules, nets, rings, plaques, and other types, which may be accompanied by ulcer, erosion, blisters, and other lesions, and (b) a pathological diagnosis of oral lichen planus, with hyperkeratosis or incomplete keratinization of the epithelium, proliferation or atrophy of the spinous layer, and liquefaction and degeneration of the basal cell layer.

Patients were excluded from the study for the following reasons: (a) taking antibiotics in the previous 3 months; (b) receiving periodontal treatment in the previous 6 months; (c) suffering other oral mucosa diseases; (d) smoking; (e) suffering systemic diseases such as diabetes and cardiovascular disease; (f) women who are pregnant, breastfeeding, menstruating, or planning to become pregnant during the study period; and (g) inability or unwillingness to sign the informed consent form.

### 2.4. Clinical Examination of Periodontal Parameters

After signing an informed consent form, oral clinical examination of the patients was performed on the first visit by the same senior examiner, with an intraindividual kappa coefficient of 0.827 (*P* < 0.001). Periodontal parameters include full-mouth probing depth (PD), clinical attachment loss (CAL), and percentage of bleeding on probing (BOP) by a UNC-15 periodontal probe (Hu-Friedy®, Chicago, USA). PD and CAL were measured at six sites per tooth and expressed by means.

### 2.5. Collection of Subgingival Plaque Samples

Patients were instructed to rinse the mouth before collecting subgingival plaque samples. After isolating the sampling sites with cotton rolls and gently drying surfaces of teeth with the air-water syringe, subgingival plaque samples were collected by a sterilized Gracey curette (Hu-Friedy®, Chicago, USA) and then placed immediately into an Eppendorf tube containing 0.5 ml of phosphate-buffered saline solution. All Eppendorf tubes were transferred to a −80°C refrigerator for preservation immediately.

### 2.6. DNA Extraction and PCR Amplification

Total genomic DNA samples of the subgingival plaque were extracted using the QIAamp DNA Mini Kit (Qiagen, Hilden, Germany) following the manufacturer's instructions. The quantity of extracted DNAs was measured using a NanoDrop ND-2000 spectrophotometer (Thermo Scientific, Wilmington, DE, USA), and the quality of DNA was checked by 1.2% agarose gel electrophoresis, respectively.

PCR amplification of bacterial 16S rRNA gene hypervariable regions (V3-V4) was performed using the forward primer 338F (5′-ACTCCTACGGGAGGCAGCA-3′) and the reverse primer 806R (5′-GGACTACHVGGGTWTCTAAT-3′). The PCR components contained 5 *μ*l of buffer (5x), 0.25 *μ*l of Fast pfu DNA Polymerase (5 U/*μ*l), 2 *μ*l (2.5 mM) of dNTPs, 1 *μ*l (10 *μ*M) of each forward and reverse primer, 1 *μ*l of DNA Template, and 14.75 *μ*l of ddH_2_O. The PCR reaction parameters were as follows: predenaturation at 98°C for 5 min; 25 cycles of denaturation for 30 s at 98°C, annealing for 30 s at 52°C, and extension for 45 s at 72°C; and a final extension for 5 min at 72°C. PCR amplicons were purified with Vazyme VAHTSTM DNA Clean Beads (Vazyme, Nanjing, China) and quantified using the Quant-iT PicoGreen dsDNA Assay Kit (Invitrogen, Carlsbad, CA, USA). The PCR product was detected by 2% agarose gel electrophoresis.

### 2.7. Bioinformatic and Statistical Analysis

After the samples were successfully amplified, they were subjected to sequencing on the Illumina MiSeq PE300 sequencing platform (Illumina, San Diego, CA, USA) with MiSeq Reagent Kit V3. Valid sequences were primarily obtained via collation and filtering of the original sequence data.

Sequence data analyses were mainly performed using QIIME2 and R packages (v3.2.0). With the QIIME software package, high-quality sequences were clustered into operational taxonomic units (OTUs) at a 3% dissimilarity level, and the longest sequence in each OTU was selected as the representative sequence. Taxonomic information of qualified sequences was obtained from the SILVA database (SILVA 132; http://www.arb-silva.de) for taxonomic analysis.

The sufficiency of the sampling effort was evaluated by rarefaction curves; the bacterial community richness and indices were estimated using the Chao1 index, Shannon Weaver index, and Simpson index. The beta-diversity was performed with QIIME2 (v1.9.1) to assess the differences of microbial communities between control and disease based on their composition. A principal coordinate analysis (PCoA) of weighted UniFrac was performed to compare the overall structure of the subgingival microbiome of all samples.

The Shapiro-Wilk test was carried out to evaluate variable normality. The normally distributed variables were described using the mean and standard deviation (SD), while the median and interquartile range (IQR) were used to describe nonnormally distributed data.

Differences in means for continuous variables were compared using Student's *t*-test, and differences in proportions were tested by the chi-squared test. The abundance of bacterial phyla and genus for each group was expressed as the percentage of total sequences, and the bacterial community structures of the two groups were further compared at the phylum and genus levels using the Mann-Whitney *U* test. The significance value was defined as *P* < 0.05.

All data were analyzed by a statistical program (SPSS Statistical for Windows, IBM Corp., Armonk, NY, USA, Version 19.0). Patients' names were hidden during data analysis. This study was performed according to the STROBE checklist.

## 3. Results

The patients' population characteristics and clinical examination results are shown in Table 1. No significant difference was found between the two groups in gender, age, and clinical examination (*P* > 0.05).

### 3.1. Overall Sequence Data

A total of 5,561,995 sequences were obtained from 39 subgingival plaque samples. 3,475,452 high-quality sequences were generated after data trimming and quality filtering.

### 3.2. Distribution of OTUs

**Figure 1:**
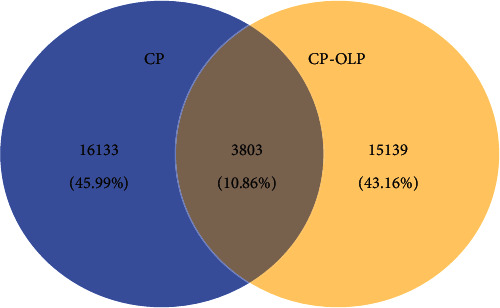
A Venn diagram showing shared and unique OTUs at 97% identity among the two groups.

OTU clustering and sequence annotation were performed using the above-obtained sequences at a 3% dissimilarity level (cutoff), and the resulting OTU tables were used for subsequent bioinformatic analysis. The OTU distributions for two groups of patients are shown in Figure 1. There were 10.86% OTUs in common between the CP and CP-OLP groups.

### 3.3. Alpha-Diversity Analysis

**Figure 2:**
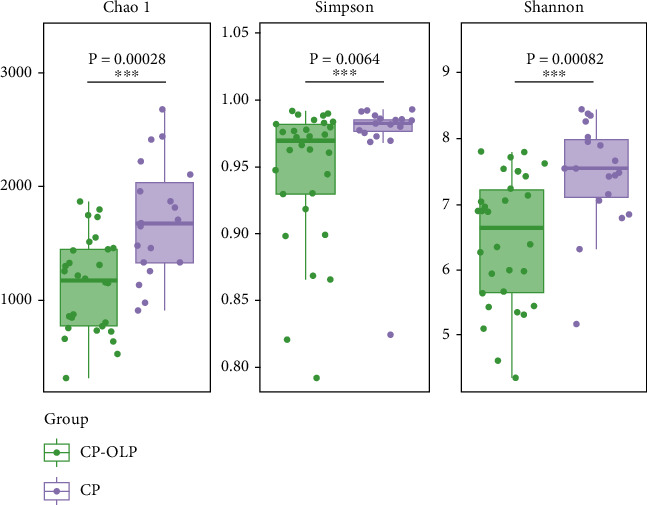
Alpha-diversity index analysis.

The average numbers of Shannon, Simpson, and Chao1 richness indices are shown in Figure 2. The results showed significantly decreased richness and diversity in the CP-OLP group compared to the CP group (*P* < 0.01).

### 3.4. Beta-Diversity Analysis

**Figure 3:**
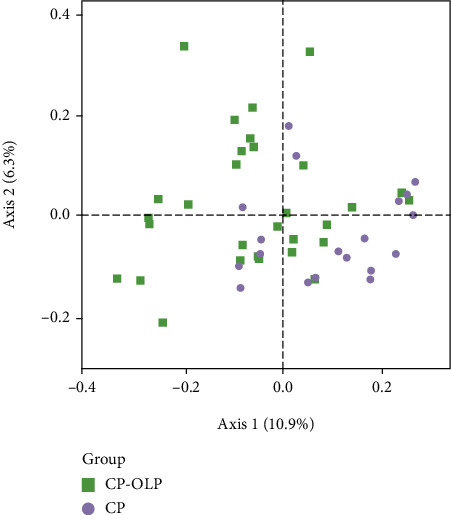
Beta-diversity visualized using PCoA. Green dots represent CP-OLP patients, and purple dots represent CP patients.

Microbial OTUs were subjected to principal coordinate analysis (PCoA) based on the weighted UniFrac distance to evaluate the similar community structures between the CP and CP-OLP groups. Samples of subgingival plaque from the CP group overlapped with some microbiota of the CP-OLP group. Axis 1 explained 10.9% of the variation, and axis 2 explained 6.3% (Figure 3).

### 3.5. Abundance Analysis

**Figure 4:**
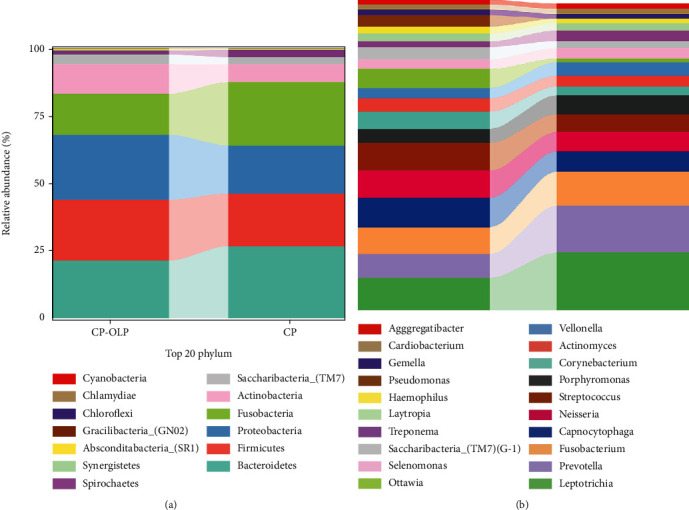
(a) Bar chart of relative abundance at phylum level; (b) bar chart of relative abundance at genus level.

The top ten dominant relative abundance ratios of the samples obtained in two groups at the phylum and genus levels are shown in Figure 4. A total of 14 bacterial phyla and 214 different genera were found in the samples obtained in two groups. *Proteobacteria*, *Bacteroidetes*, *Firmicutes*, and *Fusobacteria* were dominant phyla. And *Leptotrichia*, *Prevotella*, *Fusobacterium*, *Capnocytophaga*, *Neisseria*, *Streptococcus*, *Porphyromonas*, *Corynebacterium*, *Actinomyces*, and *Veillonella* were dominant genera. At the phylum level, the distribution of *Fusobacteria* and *Bacteroidetes* in the CP-OLP group was significantly lower than that in the CP group. As for the genus level, the distribution of *Prevotella* and *Leptotrichia* in the CP group was significantly higher than that in the CP-OLP group.

**Figure 5:**
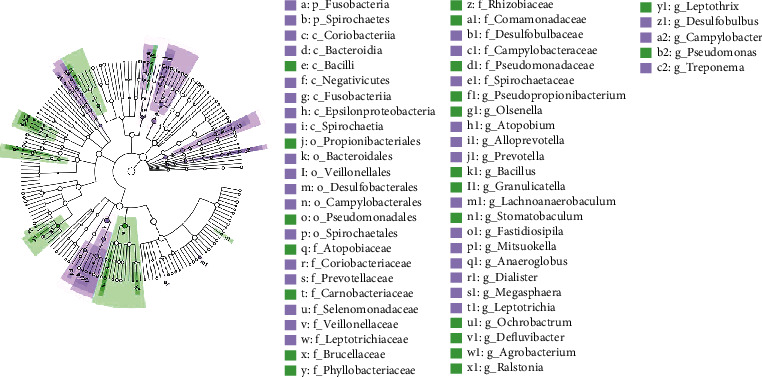
The linear discriminant analysis effect size (LEfSe).

The linear discriminant analysis effect size (LEfSe) tested the significant differences in the bacterial compositions between the two groups (Figure 5). The result showed that the dominant genus in the CP-OLP group was *Pseudomonas* and *Granulicatella*, with the abundance of *Leptotrichia* and *Prevotella* in the CP group.

## 4. Discussion

Oral lichen planus is a common chronic inflammatory oral mucosal disease, with a prevalence of 0.5-2.2% in the general adult population [13]. The two main subtypes of OLP are reticular OLP and erosive OLP. Erosive OLP is characterized by erythema, erosion, and ulcerative lesions [14]. Many patients with erosive gingival OLP usually have a complaint of “gingival bleeding” or “gingival pain,” which limits efficient tooth brushing. It results in additional plaque accumulation. Although Student's *t*-test in this study showed that there was no significant difference, all of the periodontal clinical examination results in the CP-OLP group were poorer than that in the CP group. It indicated that the OLP lesion may aggravate periodontal damage. Correspondingly, local factors, such as dental plaque and calculus, disallow the improvement or recovery of erosive gingival lesions, particularly in areas with high levels of local irritants.

Oral microbial changes in the OLP disease state are not clear, but some studies have confirmed the correlation between OLP and oral microorganisms [9]. Although the clinical types and presence or absence of symptoms were not specified, Ertugrul et al. [11] found that the proportion of *Aggregatibacter actinomycetemcomitans*, *Porphyromonas gingivalis*, *Prevotella intermedia*, *Tannerella forsythia*, and *Treponema denticola* in total bacteria were higher in subgingival plaque samples taken from subjects having periodontitis and OLP than those having periodontitis without OLP. The results of these studies suggest that there may be a correlation between the microbe and OLP. To the best of our knowledge, there is no sufficient research to show the differences in subgingival microbial flora in periodontitis patients with OLP and periodontitis patients. In this study, the subgingival plaque microflora was investigated by 16S rRNA gene high-throughput sequencing from CP patients and CP-OLP patients. The findings suggest that microbiome in erosive OLP with CP was significantly different from that found in only CP, and microbiome changes might be related to the presence or absence of OLP disease.

Oral microbiota composition has been widely analyzed in recent years [15–17]. More than 280 oral bacterial species have been isolated and characterized by using cultivation methods traditionally [18]. However, 31% of oral bacterial taxa have not been grown in vitro, which hinders the thorough and further understanding of the microbial community in oral plaque microflora [19]. Other molecular biological techniques have obvious drawbacks and shortcomings such as denaturing gradient gel electrophoresis, the quantitative real-time polymerase chain reaction, and microarray chips [20]. Currently, the 16S rRNA gene sequencing method has been used to study uncultivable species, which have been the primary basis of defining the oral microbiome and facilitated a comprehensive study of the analysis of bacterial diversity [21].

The richness and diversity of subgingival plaque microflora in the CP group increased significantly compared with the CP-OLP group. In the perspective of the oral cavity, these findings are relevant since the reduction in oral bacterial diversity is associated with a high risk of oral mucosa diseases [22]. It suggested that the change in the composition ratio of the flora breaks the balance of the original flora, thus destroying the physical barrier of the epithelium.

There is a frequent presence of bacterial and fungal opportunists in patients with oral mucosal complaints. Representative microorganisms in opportunistic infections of the oral cavity are *Pseudomonas aeruginosa*, *Staphylococcus aureus*, and *Candida albicans* [23]. Slots et al. [24] showed *Pseudomonas* in the subgingival flora of patients suffering from severe periodontitis. This was consistent with another study that found that the prevalence of opportunistic *Pseudomonas* in epithelial cells is correlated with the state of periodontal disease. In the present study, the relative abundance of *Pseudomonas* in the CP-OLP group was increased than that of the CP group, which indicated that OLP may increase susceptibility to opportunistic infections in periodontitis patients and enhance inflammation in the periodontium [25].

*Granulicatella* species are facultative anaerobic, catalase-negative Gram-positive cocci [26]. Oral microbiome research found out that *Granulicatella* species are dominant bacteria in the oral cavity. It was detected in all samples of patients with chronic periodontitis, necrotic ulcerative periodontitis, primary pulp infection, persistent pulp infection, or caries [27–29]. Du et al. [30] reported that compared with healthy controls, enrichment of *Granulicatella* was more abundant in patients with OLP. In recent years, many cases of infection caused by *Granulicatella* species have been reported. In addition to the oral diseases mentioned above, a variety of infections are also involved, including bacteremia, infective endocarditis, brain abscess, central nervous system infection, and maxillary sinusitis [31–33]. It could give a hint that this bacterium closely relates to infection. The significantly increased abundance of *Granulicatella* in the CP-OLP group in our study implies that erosive OLP lesions might increase the risk of infection at the periodontitis site.

The result of this study showed that the dominant genus in the CP group was *Leptotrichia* and *Prevotella*. It was in accordance with a previous well-known study [34]. Several documents and literature reviews have pointed out that *Leptotrichia* is an opportunistic pathogen. It could be isolated and recovered from various sources, including gingivitis, necrotizing ulcerative gingivitis, adult/juvenile periodontitis, “refractory periodontitis,” and human and animal infections [35]. Meanwhile, as a member of the orange complex, *Prevotella* is one of the most frequently encountered species in subgingival plaque. And it is considered a periodontitis-associated member of the subgingival microbiota and the major pathogen in advancing periodontitis [36]. Both of them seem to be crucial in the pathogenesis of periodontitis disease.

In our study, the number of patients in two groups is not consistent due to objective reasons (COVID-19). Since the subgingival microorganisms reflect the overall periodontal microbial environment, recording periodontal indices of all of the remaining teeth is used for statistics analysis in the study. It was known that a new classification of periodontal and peri-implant diseases and conditions was summarized and published in June 2018 [37]. It was not used in our study as clinical periodontal data collection was carried out before the publication of the new classification. In addition, our study was a single-center small sample observational study for which the small sample number may affect the results of the study. The longer-term prognosis needs further observation, for which we look forward to more large-scale and multicenter clinical study.

In summary, the bacterial diversity and variation of oral microbiota in OLP and CP patients were investigated by high-throughput sequencing. Our data indicated statistically significant changes in the subgingival plaque in the CP-OLP group compared to that in the CP group, implying a correlation between the changes in subgingival microbial composition and OLP incidence. The relationship between subgingival plaque microorganisms and OLP could be explained in two aspects. Firstly, the number and compositions of plaque microorganisms change as OLP lesions are formed. In other words, subgingival plaque as an initial factor of periodontal disease might prevent OLP gingival lesions from healing, which changes the characteristics of the lesions into more aggressive forms such as erosive lesions. Secondly, subgingival plaque microbiome plays a direct or indirect role in the development of OLP lesions and may lead to the aggravation of periodontitis and opportunistic infection. However, to date, the knowledge about the role of subgingival plaque microbiome changes in the incidence of OLP is still limited, and further research is required.

## Figures and Tables

**Table 1:** Characteristics of study participants.

Group	Age (year)	Sex (male/female)	CAL (mm)	PD (mm)	BOP (%)
CP group	45.8 ± 8.0	8/12	2.08 ± 0.55	2.14 ± 0.52	50.35 ± 18.49
CP-OLP group	49.2 ± 9.5	6/13	2.13 ± 0.55	2.39 ± 0.45	59.25 ± 16.52
*χ* ^2^		0.300			
*P*	0.734	0.584	0.720	0.086	0.094

CAL: clinical attachment level; PD: probing depth; BOP: bleeding on probing.

## Data Availability

All sequences were deposited in the NCBI Nucleotide Archive database under project no. PRJNA690677.
